# Examining a 12-year experience within Kazakhstan’s national heart transplantation program

**DOI:** 10.1038/s41598-024-61131-1

**Published:** 2024-05-04

**Authors:** Yuliya Semenova, Saule Shaisultanova, Altynay Beyembetova, Aruzhan Asanova, Aliya Sailybayeva, Svetlana Novikova, Gulzhan Myrzakhmetova, Yuriy Pya

**Affiliations:** 1 School of Medicine, Nazarbayev University, Astana, 010000 Kazakhstan; 2 Corporate Fund “University Medical Center”, Astana, 010000 Kazakhstan; 3 RSE on PCV “Republican Center for Coordination of Transplantation and High-Tech Medical Services” of the Ministry of Health, Astana, 010000 Kazakhstan

**Keywords:** Heart transplantation, Cardiovascular disease, Survival, Time series, Kazakhstan, Cardiology, Health care

## Abstract

Kazakhstan has one of the lowest heart transplantation (HTx) rates globally, but there are no studies evaluating the outcomes of HTx. This study aimed to provide a comprehensive analysis of the national HTx program over a 12-year period (2012–2023). Survival analysis of the national HTx cohort was conducted using life tables, Kaplan‒Meier curves, and Cox regression methods. Time series analysis was applied to analyze historical trends in HTx per million population (pmp) and to make future projections until 2030. The number of patients awaiting HTx in Kazakhstan was evaluated with a regional breakdown. The pmp rates of HTx ranged from 0.06 to 1.08, with no discernible increasing trend. Survival analysis revealed a rapid decrease in the first year after HTx, reaching 77.0% at 379 days, with an overall survival rate of 58.1% at the end of the follow-up period. Among the various factors analyzed, recipient blood levels of creatinine and total bilirubin before surgery, as well as the presence of infection or sepsis and the use of ECMO after surgery, were found to be significant contributors to the survival of HTx patients. There is a need for public health action to improve the HTx programme.

## Introduction

According to the universal definition of heart failure (HF), it is characterized as a clinical syndrome marked by symptoms and/or signs stemming from structural and/or functional abnormalities in the heart^1^. Recognized as a global pandemic, HF reached an estimated worldwide prevalence of 64.3 million people in 2017^2^ and is anticipated to increase due to extended life expectancy and enhanced survival of HF patients attributed to life-saving treatments^3^. The advanced stage of HF necessitates surgical intervention. Heart transplantation (HTx) is considered the gold standard among these interventions and is likely to enhance survival and improve quality of life in such patients^4^. In addition to HTx, a variety of ventricular assist devices are available, with left ventricular assist devices (LVADs) being the most commonly implanted^5^.

Despite the benefits of HTx, there is a shortage of hearts available for transplantation, and only a limited number of eligible patients receive these interventions. While there was a decline in HTx numbers from the 1990s to the early 2000s, the trend has been on the rise over the past decade^6^. According to data from the Global Observatory on Donation and Transplantation (GODT), 8988 hearts were transplanted globally in 2022, representing a 7% increase from 2021. The per million population (pmp) rates of HTx are highest in the Americas (5.1) and European region (3.5) and lowest in the African region, where they equal 0.0 in 2022^7^.

Kazakhstan, a post-Soviet country in Central Asia, has one of the lowest HTx rates globally^7^. The country initiated its first heart transplantation in 2012 and concurrently launched an LVAD implantation program. Despite an early-stage analysis of the LVAD program^8^, there is a notable dearth of studies evaluating the national HTx program. Consequently, this study aims to comprehensively analyze the national HTx program over a 12-year period (2012–2023), focusing on both its outputs and outcomes. Specifically, this study aimed to elucidate outputs by examining historical trends in HTx and the population in need. The rates of LVAD implantation will be juxtaposed with those of HTx. Future projections until 2030 will be conducted to forecast the demand for these services. Furthermore, this study intends to scrutinize the number of patients awaiting HTx in Kazakhstan, with a regional breakdown. Additionally, this study aimed to assess the outcomes of the national HTx program by conducting a survival analysis of the national HTx cohort.

## Methods

This study consists of two distinct stages: the analysis of HTx program outputs and outcomes. The primary source of data was the Republican Center for Coordination of Transplantation and High-Tech Services, hereafter referred to as the Transplantation Coordination Center, under the Ministry of Health (MoH) of Kazakhstan. Functioning as the national agency providing technical support to all hospitals performing organ transplantations in Kazakhstan, this center is responsible for maintaining the medical information system, specifically the Registry of Donors and Recipients, which encompasses data on donors and recipients for various organ transplantations.

The regulation of organ acquisition and donor management in Kazakhstan is governed by the Transplant Act. This legislation aligns with the principles outlined by the International Society for Heart and Lung Transplantation (ISHLT)^9^. According to the Act, organs can be donated following brain death by individuals aged 18 years and older, provided that they have officially confirmed their willingness for postmortem organ donation during their lifetime. This declaration of intent can be made through the "electronic government" web portal or through a general practitioner. In cases where explicit consent from the individual is not available, organ donation is contingent upon the consent of their family^10^.

Presently, four hospitals in Kazakhstan conduct HTx, with the National Scientific Cardiac Surgery Center in Astana being the primary facility for the majority of HTx and LVAD implantations nationwide. The other three hospitals undertaking these surgical interventions are the Heart Center Shymkent, the Research Institute of Cardiology and Internal Diseases, and the National Scientific Center of Surgery named after Syzganov. The operations of the Transplantation Coordination Center, including the management of the Registry of Donors and Recipients, are governed by the Ministry of Health's directive^11^.

### Analysis of the output parameters in the heart transplantation program

#### Analysis of data on HTx and LVAD implantation in Kazakhstan

The annual nationwide rates of HTx and LVAD implantation were computed per million population (pmp). This involved retrieving the number of HTx and LVAD implantations performed in Kazakhstan from the inception of these procedures in 2012 to the present year (2023) from the Registry. Population figures for Kazakhstan in the respective years were sourced from the Demographic Yearbook, a statistical compilation issued by the Bureau of National Statistics^12^. The aggregated data encompassing nationwide annual incidences of ischemic heart disease (IHD) and heart valve disease (HVD), along with the pmp rates of HTx and LVAD implantations, were organized in an Excel spreadsheet, indicating the reference year for the statistics.

#### Analysis of incidence rates of ischemic heart disease and heart valve disease

To augment the findings of our study regarding the rates of HTx and LVAD implantation and to generate forecast projections concerning future healthcare needs in the country, we obtained official statistics on the incidence rates of IHD and HVD in Kazakhstan. These two medical conditions represent the primary indications for HTx and LVAD implantation, in accordance with the national standard of care^13^. The MoH of Kazakhstan annually publishes a yearbook of national health statistics^14^. For our research, we retrieved data on the incidence of IHD and HVD spanning the years 1998 to 2022 from this resource. The electronic versions of these yearbooks are freely accessible on the website of the National Research Center for Health Development named after Salidat Kairbekova, and details on studies conducted utilizing this yearbook's information are available elsewhere^15^.

We downloaded yearbooks covering the period from 1998 to 2022 and extracted precalculated incidence rates of IHD and HVD from the "Health Indicators" subsection. The incidence of IHD specifically pertains to adults, defined as individuals aged 18 years and older, and is presented per 100,000 people. Similarly, the incidence of HVD is based on the total population, encompassing both adults and children, and is also presented per 100,000 people.

#### Time series analysis

The Expert Modeler function of SPSS was employed to automatically identify the best-fit epidemiological models for each type of predictive analysis: incidence rates of IHD and HVD, as well as pmp rates of HTx and LVAD implantation until 2030. The observed incidences from 1998 to 2022 were utilized for IHD and HVD, while the observed rates for HTx and LVAD implantation from 2012 to 2023 were employed. All projections were reported as estimates along with their 95% confidence intervals (CIs), and corresponding graphs were generated. The significance level for the best-fit epidemiological models was set at 0.05.

#### Geospatial analysis

The cumulative figures of all adult and pediatric patients awaiting heart transplantation across Kazakhstan's regions from the inception of the program to the end of 2023 were entered into an Excel spreadsheet. The population data for each region of Kazakhstan were acquired from the website of the Bureau of National Statistics to calculate the pmp rates^16^. To visualize regional variations in the rates of patients awaiting HTx, the Quantum Geographic Information System (QGIS) Version 3.26 "Buenos Aires" was used.

### Analysis of the outcome parameters in the heart transplantation program

Survival analysis was conducted to evaluate the outcome parameters of the national HTx program. Data on all HTx patients in Kazakhstan were acquired in a fully anonymized manner from the Transplantation Coordination Center. The dataset retrieved from the Registry for HTx patients included various details, such as the date of the HTx procedure, date of death (if applicable), age and sex, blood group and rhesus D (RhD) factor, as well as the weight and height of both the donor and recipient. The inclusion of the latter two variables facilitated the calculation of body mass index (BMI) using the following formula: weight in kilograms divided by height in meters squared. Additionally, information on the cause of death of heart donors was obtained from the registry. Currently, brain death serves as the sole parameter for organ acquisition^10^, and the causes of donor death are categorized into four broad groups: ischemic stroke, hemorrhagic stroke, brain injury, and neoplasm.

For heart recipients, more extensive data were available, including laboratory values before HTx surgery, such as hemoglobin, creatinine, glomerular filtration rate (GFR), total bilirubin, lactate dehydrogenase, sodium, potassium, white blood cell count, and C-reactive protein. Information related to previous cardiac surgeries, as well as coexisting pathologies such as arterial hypertension, hypothyroidism, pulmonary hypertension, and the type of cardiomyopathy, was also obtained from the registry. In addition, information on the length of postoperative hospital stay and postsurgical morbidity [infection/sepsis, rejection within hospital stay, hemodialysis, resternotomy, and extracorporeal membrane oxygenation (ECMO)] was available and extracted from the registry.

Life tables, Kaplan‒Meier analysis (K‒M analysis), and Cox regression were the three survival analysis methods employed in this study. The data sourced from the registry were organized in Excel spreadsheets, with the primary variables being the date of HTx, the date of death, or the conclusion of the follow-up period (30th November 2023). Given the unavailability of precise causes of death for HTx patients, overall survival was reported.

Life tables were generated to estimate cumulative survival at specific time intervals: 30 days, 90 days, 180 days, 360 days, 720 days, 1080 days, 1380 days, and 1740 days. The number of patients who survived and those who died at the end of each interval was documented. Cumulative mortality rates were calculated using the following formula: 100 − cumulative survival.

K‒M analysis was employed to assess the probability of surviving until the end of the follow-up, along with the mean and median survival of HTx patients, presented with 95% CIs. Since the cumulative survival at the end of the follow-up was 58.1%, only the mean survival time was reported. A graph depicting the overall survival curve during the study period was generated.

Multivariate Cox regression analysis was employed to assess the risk factors associated with mortality among HTx patients. The selection of factors was based on their independence from each other. These factors were categorized into two main groups, donor-related and recipient-related, and their respective effects were reported separately. The donor-related factors included age, sex, weight, height, blood group, RhD factor, and cause of death. Recipient-related factors were further subdivided into three categories: presurgical factors (such as age, sex, weight, height, blood group, RhD factor, history of previous cardiac surgeries including LVAD implantation, arterial hypertension, hypothyroidism, pulmonary hypertension, and type of cardiomyopathy—ischemic, dilated, or valvular), presurgical laboratory values (including hemoglobin levels (g/L), creatinine levels (mg/dL), GFR (mL/min), total bilirubin levels (mg/dl), lactate dehydrogenase levels (U/L), sodium levels (mmol/L), potassium levels (mmol/L), white blood cell count, and C-reactive protein levels (mg/L)), and postsurgical factors (such as length of postoperative hospital stay and postsurgical morbidity including infection/sepsis, rejection within hospital stay, requirement for hemodialysis, resternotomy, and ECMO). Adjusted hazard ratios (HRs) were computed for all variables, and the statistical significance level of the Cox regression model was set at 0.05. Survival analysis was performed using the "Survival" function in the Statistical Package for Social Sciences (SPSS) version 24.0 for Windows.

### Ethics declaration

This study was conducted in strict accordance with the principles outlined in the Helsinki Declaration. Only completely anonymized data obtained from the Transplantation Coordination Center were analyzed. Prior to commencing the data collection, approval was obtained from the Local Commission on Bioethics of the Corporate Fund “University Medical Center” (hereafter referred to as the Ethics Committee). The Ethics Committee reviewed the case and waived the need for informed consent, as documented in the Minutes of the meeting of Ethics Committee #3 dated July 14, 2023.

## Results

### Output study

**Figure 1:**
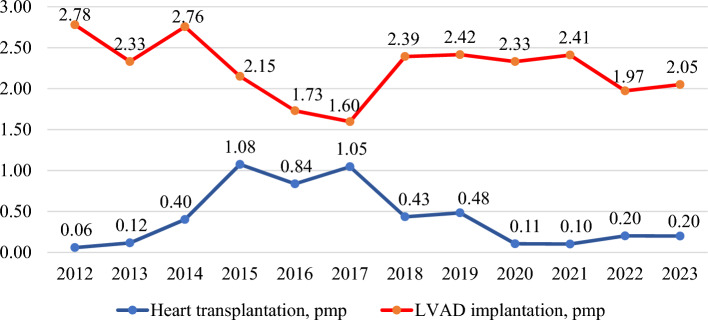
Rates of heart transplantation and left ventricular assist device (LVAD) implantation per million population (pmp), 2012–2023.

The first HTx and LVAD implantation in Kazakhstan occurred in 2012. Over the period of 2012–2023, 92 HTx and 493 LVAD implantations were performed. Specifically, there was one HTx in 2012, two in 2013, seven in 2014, nineteen in 2015, fifteen in 2016, nineteen in 2017, eight in 2018, nine in 2019, two in 2020, two in 2021, four in 2022, and four in 2023. Notably, the peak number of HTx procedures occurred between 2015 and 2017, while the lowest number of LVAD implantations was observed during the same period. Figure 1 illustrates a declining trend in the pmp rates of HTx and LVAD patients. The percentage of annual decline for HTx was 2.03% (95% CI, from − 19.11 to 18.66%, p = 0.408), and for LVAD implantation, it was 1.39% (95% CI, from − 4.44 to 1.76%, p = 0.173).

**Figure 2:**
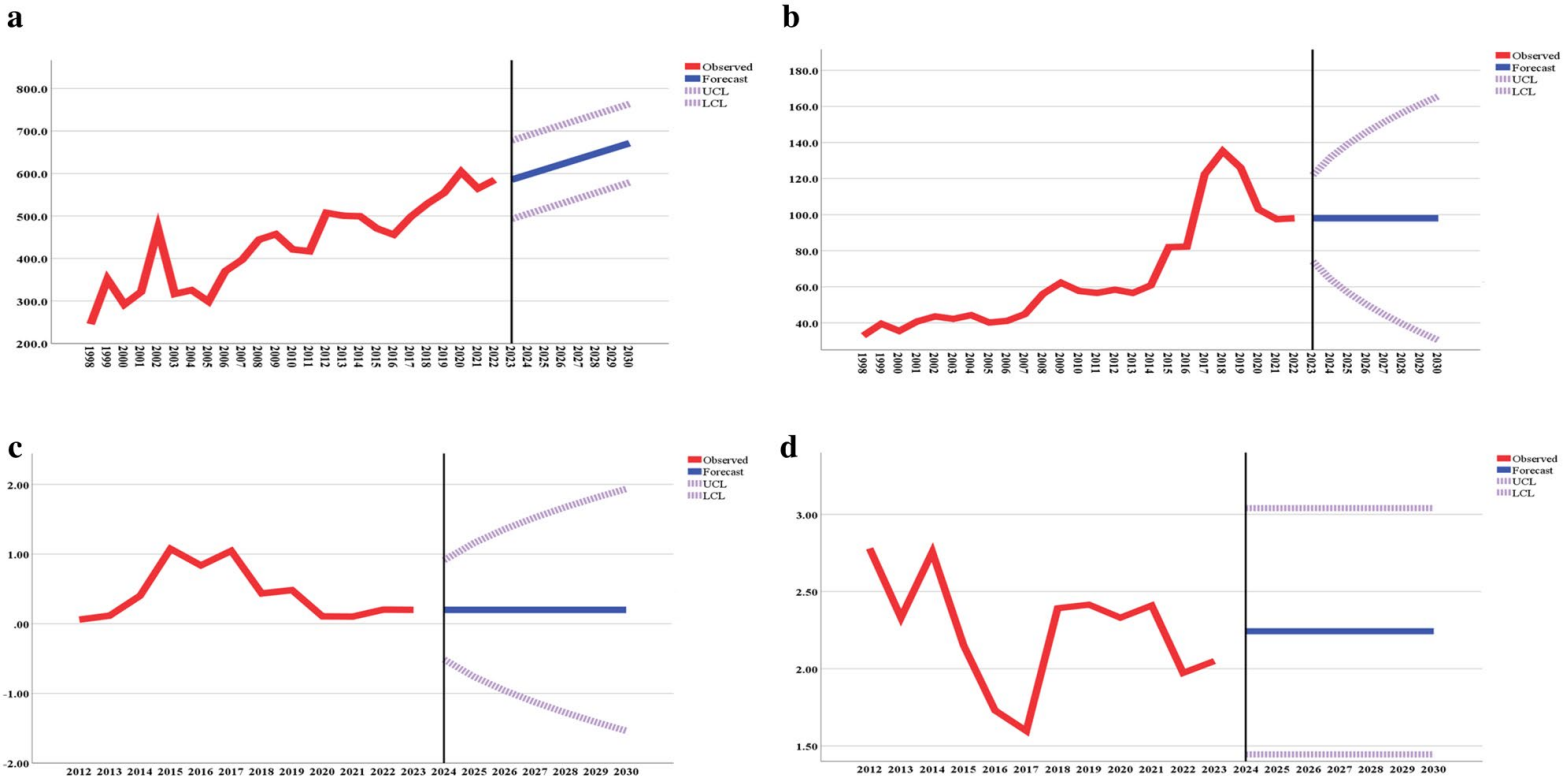
The observed and projected rates of ischemic heart disease (**a**), heart valve disease (**b**), heart transplantation (**c**) and left ventricular assist device implantation (**d**) until 2030.

Table 1 provides estimates of the projected incidence rates of IHD and HVD, the major indications for HTx according to national standards of care^13^. The table also presents estimates of the projected per capita rates of HTx and LVAD implantation. The rate of IHD is projected to increase to 610.0 per 100,000 people (95% CI from 517.7 to 702.4) in 2025 and to increase to 671.3 (95% CI from 579.0 to 763.7) in 2030. However, according to projections, no growth is expected for HTx and LVAD implantations. Figure 2 indicates a steady increase in IHD incidence from 1998 to 2022, with this trend expected to continue. Conversely, the trends for HTx and LVAD implantation appeared stable.

**Table 1:** The projected rates of ischemic heart disease, heart valve disease, heart transplantation, and left ventricular assist device (LVAD) implantation in Kazakhstan for the years 2025 and 2030, accompanied by 95% confidence intervals (CIs).

Parameter	Year		Model parameters		
	2025 rate (95% CI*)	2030 rate (95% CI*)	Type of model	Alpha (level)	
				t	P value
Incidence rates, per 100,000 population					
Ischemic heart disease	610.0 (517.7–702.4)	671.3 (579.0–763.7)	Holt	0.28	0.978
Heart valve disease	98.0 (56.6–139.4)	98.0 (30.4–165.6)	Simple	4.932	< 0.0001
Surgeries per million population					
Heart transplantation	0.20 (− 0.76–1.16)	0.20 (− 1.54–1.94)	Simple	3.045	0.011
LVAD** implantation	2.24 (1.45–3.04)	2.24 (1.45–3.04)	ARIMA (0,0,0)	2.243	< 0.0001

*95% confidence interval.

**Left ventricular assist device.

**Figure 3:**
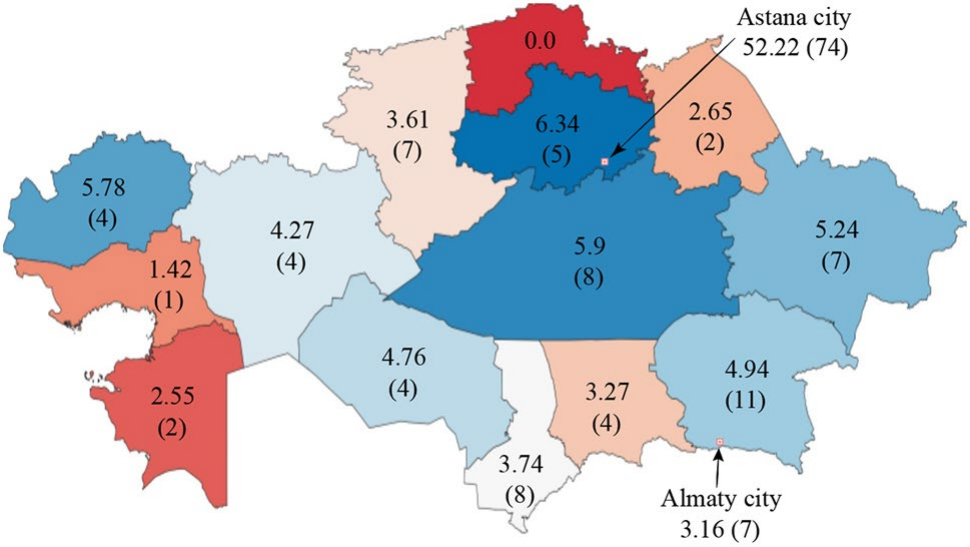
A map illustrating the distribution of patients awaiting heart transplantation across Kazakhstan's regions from the inception of the program to the end of 2023, indicating the number per million population and the total number (in brackets). The map was produced utilizing Quantum Geographic Information System (QGIS) software, Version 3.26 'Buenos Aires'" (https://www.qgis.org/en/site/).

From the inception of the HTx program to the end of 2023, 146 patients, comprising 139 adults and 7 children, awaited HTx in Kazakhstan. The Akmola, Karaganda, and West Kazakhstan regions had the highest per capita percentages of patients waiting for HTx (6.34, 5.90, and 5.78 pmp, respectively). In the North Kazakhstan region, there were no patients on the waiting list. However, Astana city had the highest percentage of patients waiting for HTx—52.22 pmp—as the majority of HTx procedures are performed there (Fig. 3).

### Outcome study

Table 2 shows the characteristics of the HTx donors and recipients. The median age of the donors was 43.0 years, while the median age of the recipients was 41.5 years. Given the policy in Kazakhstan, which prohibits children from being donors, there were no donors below the age of 18, although two recipients fell within that age group. The predominant age category for both donors (42.0%) and recipients (28.6%) was 30–50 years. None of the donors were underweight (defined as having a BMI < 18.5), while 1.8% of the recipients fell into this category. Generally, the median BMI of the donors was greater than that of the recipients (24.2 vs. 23.3). Blood group 0 predominated among donors (35.2%), whereas blood group A was most common among recipients (35.6%). The prevalence of negative RhD was lower among recipients than among donors (4.5% vs. 13.6%).

**Table 2:** Characteristics of a national cohort of heart donors and recipients, 2012–2023 (n = 88).

Characteristic	Donors N (%)	Recipients N (%)
Median age (Q1–Q3), years	43.0 (32.0–52.0)	41.5 (27.5–54.0)
Age group, years		
< 18	0 (0.0)	2 (1.8)
18–29	16 (14.3)	23 (20.5)
30–50	47 (42.0)	32 (28.6)
51–65	23 (20.5)	31 (27.7)
> 65	26 (23.2)	24 (21.4)
Sex		
Female	36 (40.9)	16 (18.2)
Male	52 (59.1)	72 (81.8)
Median body mass index (Q1–Q3)	24.2 (22.3–25.9)	23.3 (21.5–26.1)
Body mass index by group		
< 18.5	0 (0.0)	2 (1.8)
18.5–24.9	61 (54.5)	56 (50.0)
25.0–29.9	19 (17.0)	25 (22.3)
≥ 30.0	32 (28.6)	29 (25.9)
Blood group		
0	31 (35.2)	16 (18.4)
A	28 (31.8)	31 (35.6)
B	22 (25.0)	27 (31.0)
AB	7 (8.0)	13 (14.9)
Rhesus D antigen		
Positive	76 (86.4)	84 (95.5)
Negative	12 (13.6)	4 (4.5)

Survival analysis data were available for 88 of the 92 HTx patients. Table 3 presents the life table with survival and mortality rates for patients who underwent HTx. The 30-day cumulative survival rate was 94%, and the 360-day survival rate was 76%. The 1080-day (3-year) survival rate was 71%, and the 1740-day (5-year) survival rate was 65%. Correspondingly, the 30-day cumulative mortality was 6%, increasing to 24% at 360 days, 29% at 1080 days, and 35% at 1740 days.

**Table 3:** Life table showing cumulative survival and mortality in 88 patients who underwent heart transplantation.

Interval end time, days	Number of patients survived	Cumulative survival, %	Number of patients died	Cumulative mortality, % (100 − survival)
30	83	94	5	6
90	77	84	6	16
180	73	84	4	16
360 (1 year)	67	76	6	24
720 (2 years)	60	72	7	28
1080 (3 years)	57	71	3	29
1380 (4 years)	54	67	3	33
1740 (5 years)	49	65	5	35

**Figure 4:**
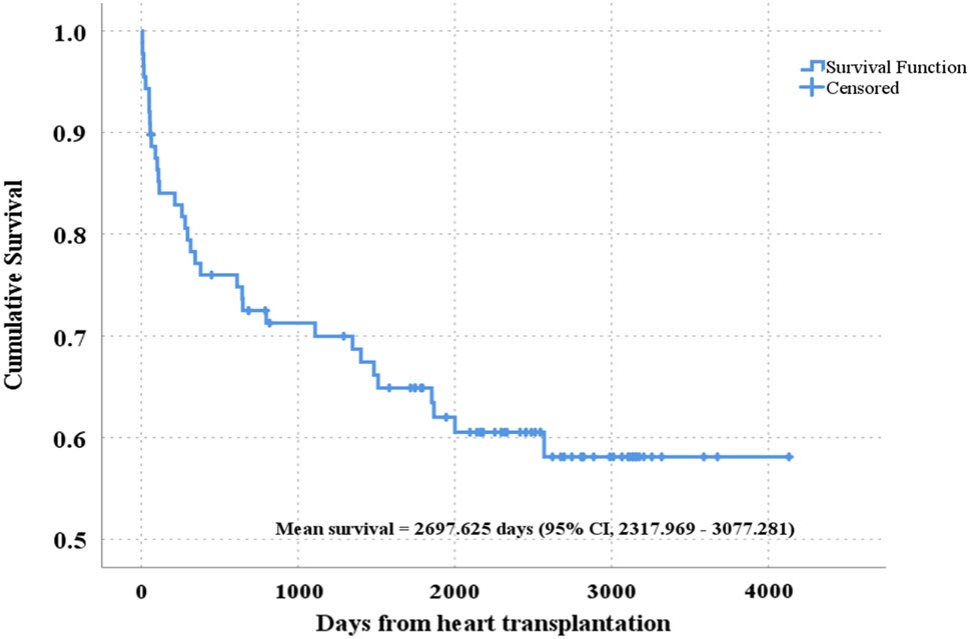
Overall survival of patients who underwent heart transplantation (n = 88).

The total duration of follow-up for the HTx patients was 4,131 days. The first year after HTx, the survival rate decreased the most rapidly, dropping to 90.9% at 55 days, 85.2% at 110 days, and 77.0% at 379 days. From day 2570 until the end of the follow-up period, the cumulative survival rate remained at 58.1% (Fig. 4).

Cox regression analysis was utilized to evaluate the hazard ratio (HR) of lethal outcomes in HTx patients based on various influencing factors pertaining to both donors and recipients. None of the donor-related factors investigated demonstrated a significant impact on lethal outcomes, as indicated in Table 4. Conversely, recipient-related factors, such as blood levels of creatinine and total bilirubin, were found to significantly influence overall survival (HR 8.979 and 7.972, respectively). Additionally, postoperative risk factors, including the presence of infection or sepsis, as well as the application of ECMO, significantly affected overall survival (HR 9.463 and 3.491, respectively), as shown in Table 5.

**Table 4:** Donor-related risk factors associated with a lethal outcome (n = 88).

Factor	Hazard ratio	P value
Age, years	1.472	0.225
Sex	0.117	0.732
Body mass index	0.364	0.546
Rhesus D antigen	0.091	0.763
Blood group	0.825	0.364
Cause of death	2.495	0.114

**Table 5:** Recipient-related risk factors associated with a lethal outcome (n = 88).

Factor	Hazard ratio	P value
Risk factors before heart transplantation		
Age, years	2.566	0.109
Sex	1.577	0.209
Body mass index	0.200	0.655
Rhesus D antigen	0.004	0.953
Blood group	0.444	0.505
Cardiac reoperation	2.958	0.085
Ventricular assist device implantation	0.181	0.670
Diabetes mellitus	0.008	0.930
Hypothyroidism	2.846	0.092
Arterial hypertension	2.393	0.122
Ischemic cardiomyopathy	0.029	0.864
Dilated cardiomyopathy	0.315	0.575
Valvular cardiomyopathy	0.005	0.946
Pulmonary hypertension	2.046	0.153
Laboratory values before heart transplantation		
Hemoglobin (g/L)	0.006	0.937
Creatinine (mg/dL)	8.979	0.003
Glomerular filtration rate (mL/min)	0.181	0.670
Total bilirubin (mg/dL)	7.972	0.005
Lactate dehydrogenase (U/L)	0.802	0.370
Sodium (mmol/L)	0.003	0.958
Potassium (mmol/L)	0.373	0.541
White blood cell count	1.035	0.309
C-reactive protein (mg/L)	0.000	0.996
Risk factors after heart transplantation		
Postoperative hospital stay, days	0.009	0.954
Infection/sepsis	9.463	0.003
Rejection within hospital stay	0.340	0.560
Hemodialysis	0.122	0.727
Re-sternotomy	1.118	0.290
Extracorporeal membrane oxygenation	3.491	0.004

## Discussion

This study aimed to conduct a comprehensive analysis of the national HTx program in Kazakhstan spanning 12 years (2012–2023). The pmp rates of HTx ranged from 0.06 to 1.08, with no discernible increasing trend. Notably, the pmp rates of LVAD implantations were comparatively greater, fluctuating between 1.60 and 2.78. These rates mirrored the HTx figures, with periods of elevated HTx rates corresponding to decreased LVAD implantation rates. Forecast analysis indicated that without intervention, the expected incidence of HTx will not increase until 2030, despite a rise in the incidence of IHD, a major indication for HTx according to national standards of care. Survival analysis revealed a rapid decrease in the first year after HTx, reaching 77.0% at 379 days, and an overall survival rate of 58.1% at the end of the follow-up period. Among the various factors analyzed, recipient blood levels of creatinine and total bilirubin before surgery, as well as the presence of infection or sepsis and the use of ECMO after surgery, were found to be significant contributors to the survival of HTx patients. A detailed discussion of these findings is warranted.

In general, the pmp rates of HTx in Kazakhstan remained low, comparable to those observed in the Southeast Asian region during 2020–2023, where they equaled 0.2 in 2022^7^. Among former Soviet Union countries, Kazakhstan and Ukraine exhibited similarly low HTx pmp rates (0.2). In contrast, other post-Soviet countries with available data reported higher HTx pmp rates: Belarus (5.6), Lithuania (3.7), Russia (1.7), and Latvia (1.1). Notably, Slovenia, the Czech Republic, and Croatia, which are also former Socialist block members, boasted some of the highest HTx pmp rates (11.4, 6.7, and 6.1, respectively, in 2020)^7^.

The HTx rate per million people is influenced by multiple factors, including the number of heart transplant centers per million inhabitants. As of the end of 2023, Kazakhstan had four heart transplant centers for a population of 20 million, resulting in an indicator of 0.2. This pmp rate of heart transplant centers in Kazakhstan is among the lowest globally compared to other regions: Eastern Mediterranean (22.1), Western Pacific (11.6), Southeast Asia (9.8), the Americas (2.9), and Europe (4.4). Only the African region exhibited a lower rate, with no registered heart transplant centers in 2022^16^.

However, a key factor contributing to the low HTx pmp rate in Kazakhstan is the high rate of refusals for deceased donations. Among 47 countries reporting refusal rates, Kazakhstan ranks 4th, with a rate approaching 90%^7^. Families refusing to donate organs to deceased relatives cited various reasons, including denial of brain death, belief in a miracle, and fear of organ trade or unknown organ destinations^17^, with such fears being particularly pronounced in Kazakhstan. An incident in 2017–2018 involving accusations of Kazakhstani transplant surgeons engaging in the organ trade further heightened these fears, resulting in a lasting negative impact on organ transplantation rates in the country^18^.

The survival rates observed in this study for HTx patients align with international reports. However, it is important to note that our findings are based on a relatively small patient cohort, limiting the generalizability of our results. In the USA, the 10-year survival rate of HTx recipients who underwent surgery between 1990 and 2007 was reported to be 53%^19^. Italy reported that the overall survival of HTx patients who underwent surgery between 2012 and 2020 was 81.2% at 1 year and 79.6% at 8 years^20^. In China, the 1-year survival rate for HTx patients is 90.1%, with a 5-year survival rate of 84.0%^21^. The Registry of the International Society for Heart and Lung Transplantation reported global 1-year and 5-year survival rates of approximately 85% and 72.5%, respectively^22^. Global survival after HTx has improved in recent years, attributed to advances in medical care, although rates differ by region, with higher survival in North America than in Europe and other regions, possibly due to higher rates of heart transplantation from older donors^23^.

Apart from older donor age, various factors influence patient survival post-HTx. A study using data from the Spanish Heart Transplant Registry identified recipient age as a significant factor contributing to overall HTx patient survival^24^. Older recipient age is associated with multiple comorbidities and poor overall survival, necessitating careful patient selection^23^. Other risk factors include congenital heart disease (HR 1.65, 95% CI from 1.32 to 2.06), retransplantation (HR 1.50, 95% CI from 1.22 to 1.85), and restrictive cardiomyopathy (HR 1.36, 95% CI from 1.11 to 1.68)^22^. The diminished survival in these patient categories may be explained by an unfavorable immunological profile and suboptimal technical operative conditions, predisposing patients to early graft failure. In our study, recipient age was insignificant risk factor. Obtaining additional data, which may occur with increased HTx procedures in Kazakhstan, may reveal additional findings.

The incidence of CVD is increasing in Kazakhstan^25^. Despite efforts to enhance care quality^26^, there are disparities between regions, with rural populations having a lower CVD identification rate^14^, contributing to a greater burden of end-stage disease. This study revealed that 146 patients were awaiting HTx in 2023, and 14 others died without receiving it. With only 4 HTx procedures performed in the country during the same period, the rate of receiving HTx was 2.5% (4/160). International studies reported higher rates of patients awaiting HTx who actually received it: 66.7% in the USA^27^, 50% in the Netherlands^28^, and 41% in Iran^29^. It is evident that much needs to be done in Kazakhstan to increase the proportion of patients on the HTx waiting list who receive HTx.

This study has several limitations. The national cohort of HTx patients is relatively small, preventing robust conclusions on HTx survival rates and associated factors. However, data on all patients who underwent HTx in the country from the inception of the HTx program (2012) to the present (2023) were available, allowing meaningful observations on necessary improvements. Another limitation is that the National Registry of Donors and Recipients lacks many specific details related to donors and recipients, thereby limiting the analysis of associated risk factors. Additionally, data on the survival of patients who underwent LVAD implantation were not available, despite an earlier report on this issue^8^. We utilized the incidence rates of IHD to predict future trends and support the determination of the need to increase the rate of HTx in Kazakhstan. The data on end-stage heart failure were not available to us, and in addition to IHD, other disorders contribute to this condition. Given the many advances in the medical management of IHD^30^, fewer patients will reach the end stage and require HTx. Nevertheless, our projections show that there is no upward trend of HTx in Kazakhstan, necessitating public health action to improve this situation.

## Data Availability

The data used and analyzed during the current study are available from the corresponding author upon reasonable request from the corresponding author.

## Abbreviations

BMI: Body mass index
CI: Confidence intervals
CVD: Cardiovascular disease
GODT: Global observatory on donation and transplantation
HF: Heart failure
HR: Hazard ratio
HTx: Heart transplantation
HVD: Heart valve disease
IHD: Ischemic heart disease
LVAD: Left ventricular assist device
MoH: Ministry of health
pmp: Per million population
RhD: Rhesus D antigen
SPSS: Statistical package for social sciences
The USSR: The union of the soviet socialist republic
WHO: World Health Organization
